# Supplementary material for: A Novel TLR4-SYK Interaction Axis Plays an Essential Role in the Innate Immunity Response in Bovine Mammary Epithelial Cells
Source: Biomedicines. 2022 Dec 30;11(1):97. doi: 10.3390/biomedicines11010097 (PMC9855420; doi:10.3390/biomedicines11010097)

The flow chart of identifying pathogenic bacteria

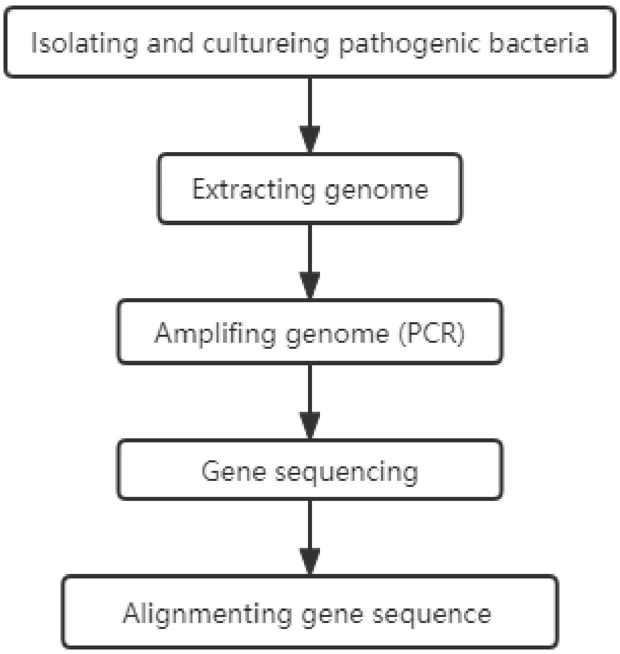

Gene sequence alignment website:
https://blast.ncbi.nlm.nih.gov/Blast.cgi?PROGRAM=blastn&PAGE_TYPE=BlastSearch&LINK_LOC=blasthome

GBS sequence
AGGGGGGGGGTAACGGCGATTACTAGCGATTCCGACTTCATGTAGGCGAGTTGCAGCCTACAA
TCCGAACTGAGATTGGCTTTAAGAGATTAGCTTGCCGTCACCGGCTTGCGACTCGTTGTACCAA
CCATTGTAGCACGTGTGTAGCCCAGGTCATAAGGGGCATGATGATTTGACGTCATCCCCACCTT
CCTCCGGTTTATTACCGGCAGTCTCGCTAGAGTGCCCAACTTAATGATGGCAACTAACAATAGG
GGTTGCGCTCGTTGCGGGACTTAACCCAACATCTCACGACACGAGCTGACGACAACCATGCAC
CACCTGTCACTTCTGCTCCGAAGAGAAAGCCTATCTCTAGGCCGGTCAGAAGGATGTCAAGACC
TGGTAAGGTTCTTCGCGTTGCTTCGAATTAAACCACATGCTCCACCGCTTGTGCGGGCCCCCGTC
AATTCCTTTGAGTTTCAACCTTGCGGTCGTACTCCCCAGGCGGAGTGCTTAATGCGTTAGCTGCG
GCACTAAGCCCCGGAAAGGGCCTAACACCTAGCACTCATCGTTTACGGCGTGGACTACCAGGG
TATCTAATCCTGTTTGCTCCCCACGCTTTCGAGCCTCAGCGTCAGTTACAGACCAGAGAGCCGCT
TTCGCCACCGGTGTTCCTCCATATATCTACGCATTTCACCGCTACACATGGAATTCCACTCTCCC
CTTCTGCACTCAAGTCCTCCAGTTTCCAAAGCGTACAATGGTTAAGCCACTGCCTTTAACTTCAG
ACTTAAAGAACCGCCTGCGCTCGCTTTACGCCCAATAAATCCGGACAACGCTCGGGACCTACGT
ATTACCGCGGCTGCTGGCACGTAGTTAGCCGTCCCTTTCTGGTTAGTTACCGTCACTTGGTAGAT
TTTCCACTCCTACCAACGTTCTTCTCTAACAACAGAGCTTTACGATCCGAAAACCTTCTTC
ACTCACGCGGCGTTGCTCGGTCAGACTTCCGTCCATTGCCGAAGATTCCCTACTGCTGCCTCCC
GTAGGAGTCTGGGCCGTGTCTCAGTCCCAGTGTGGCCGATCACCCTCTCAGGTCGGCTATGTAT
CGTCGCCTTGGTGAGCCTTTACCTCACCAACTAGCTAATACAACGCAGGTCCATCTCACAGTGA

AGCAATTGCTCCTTTTAAATAACTAACATGTGTTAATTACTCTTATGCGGTATTAGCTATCGTTTC
CAATAGTTATCCCCCGCTATGAGGCAGGTTACCTACGCGTTACTCACCCGTTCGCAACTCAT
CAGTCTAGTGTAAACACCAAACATCAGCGTTCTACTGCATGTATAGCCCCCTCC

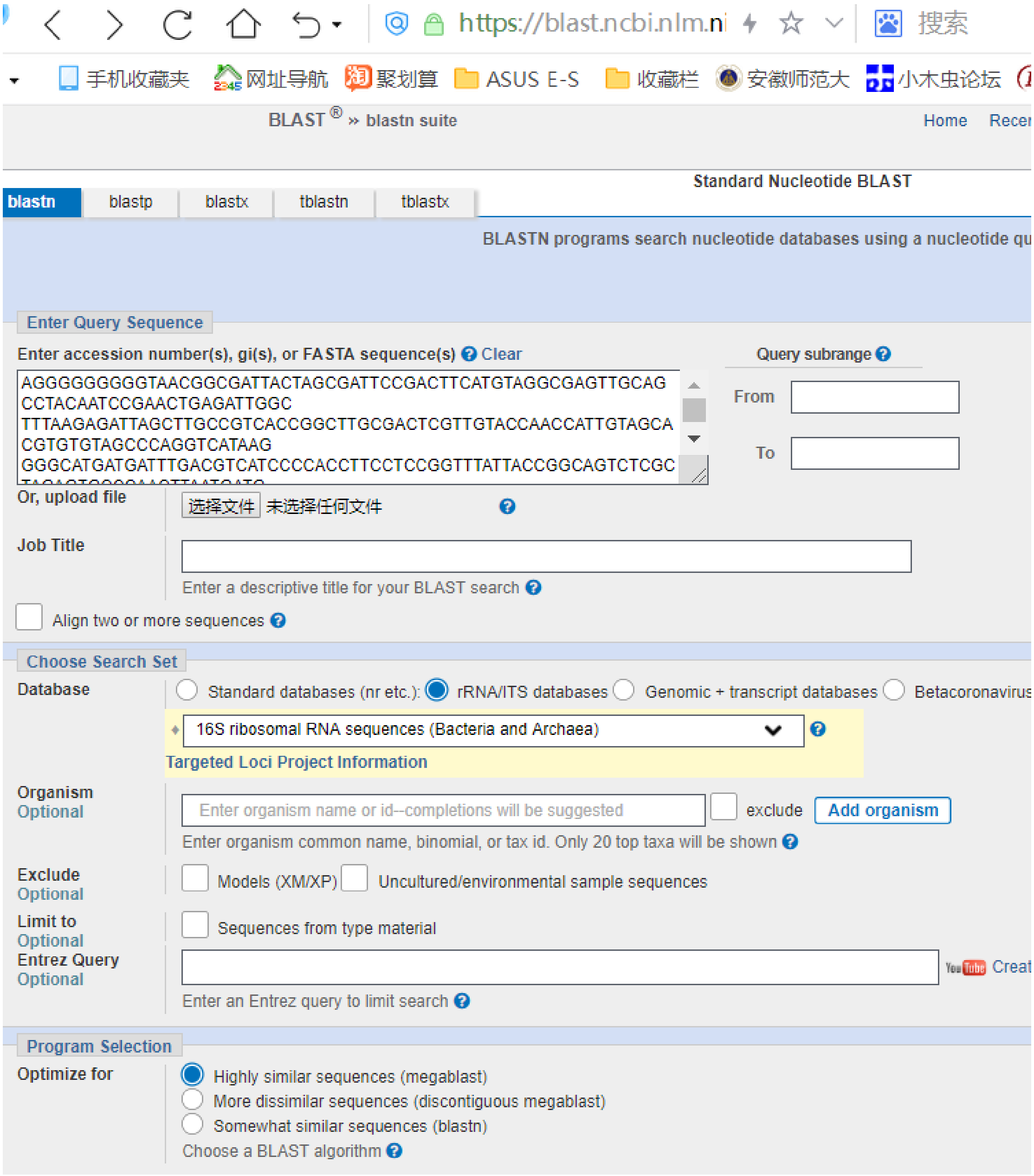

Supplement: Supplementary file 1 [file biomedicines-11-00097-s001.zip › Supplemental File S1.pdf]
