# Supplementary material for: A Novel TLR4-SYK Interaction Axis Plays an Essential Role in the Innate Immunity Response in Bovine Mammary Epithelial Cells
Source: Biomedicines. 2022 Dec 30;11(1):97. doi: 10.3390/biomedicines11010097 (PMC9855420; doi:10.3390/biomedicines11010097)
Supplement: Supplementary file 1 [file biomedicines-11-00097-s001.zip › Supplemental Table S1.pdf]

Primer name	Primer Sequence (5’-3’)	Fragments Size (bp)	Annealing Temperature (℃)
mβ-Actin-F	CCTCACGGAACGTGGTTACA	87	57
mβ-Actin-R	TCCTTGATGTCACGCACAATTT		
mSYK-F	ACAAGGACAAAACGGGGAAG	258	59
mSYK-R	TGGCTCGTAAGGGTTGAATG		
mTLR2-F	CAATCCAGAACATTAGCCATC	200	58
mTLR2-R	GTCACAGCGGTAGTCATC		
mTLR4-F	CAGGGCAGGGAAAGTCAA	203	58
mTLR4-R	AGGAAAAGTGAGCCAAGACC		
mIL1β-F	CAACCGTACCTGAACCCAT	231	59
mIL1β-R	GCCACGATGACCGACACCA		
mIL8-F	CTGCCTAAACCCCAAGGAA	206	59
mIL8-R	AACCCTACACCAGACCCACA		
mIL-10-F	GTATCCACTTGCCAACCA	237	59
mIL-10-R	CGTGCTCCTTGATGTCAG		
mAKT1-F	CCTCCTGAAGAACGATGG	148	57
mAKT1-R	GCGGATGATGAAGGTGTT		
mNLRP3-F	GGAGGAAGAAGAGGAGGAA	254	59
mNLRP3-R	CCAACCACAATCTACGAATG		
mNF-κB-F	CAGTATGCCATTGTGTTCC	152	57
mNF-κB-R	TCCTCCTTGTCTTCTACCA		

Prepare the following mixture in the qPCR tube

2 × Taq Pro Universal SYBR qPCR Master Mix	10.0 μl
Primer1 (10 μM)	0.4 μl
Primer2 (10 μM)	0.4 μl
Template DNA/cDNA	2.0μl
ddH2O	7.2μl

Amplification curve

Stage1	Predenaturation	Reps:1	95°C	30 sec
Stage2	Cyclic reaction	Reps:40	95°C	10 sec
			60°C	30 sec
Stage3	Dissociation Curve	Reps:1	95°C	15 sec
			60°C	60 sec
			95°C	15 sec
