# Supplementary material for: A Novel TLR4-SYK Interaction Axis Plays an Essential Role in the Innate Immunity Response in Bovine Mammary Epithelial Cells
Source: Biomedicines. 2022 Dec 30;11(1):97. doi: 10.3390/biomedicines11010097 (PMC9855420; doi:10.3390/biomedicines11010097)
Supplement: Supplementary file 1 [file biomedicines-11-00097-s001.zip › Supplemental Table S2.pdf]

siRNA	Sequences (5’-3’)
**siRNA-NC**	Sense: UUC UCC GAA CGU GUC ACG UTT<br>Antisense: ACG UGA CAC GUU CGG AGA ATT
**SYK-siRNA-1**	Sense: GGG AUG AAU CUG AGC AGA UTT<br>Antisense: AUC UGC UCA GAU UCA UCC CTT
**SYK-siRNA-2**	Sense: GCG GGC AAU CGG GAA AUA UTT<br>Antisense: AUA UUU CCC GAU UGC CCG CTT
**SYK-siRNA-3**	Sense: GCA GUG AAG UUU CUG CUA UTT<br>Antisense: AUA GCA GAA ACU UCA CUG CTT
