# Supplementary figures and images for: A Novel TLR4-SYK Interaction Axis Plays an Essential Role in the Innate Immunity Response in Bovine Mammary Epithelial Cells
Source: Biomedicines. 2022 Dec 30;11(1):97. doi: 10.3390/biomedicines11010097 (PMC9855420; doi:10.3390/biomedicines11010097)

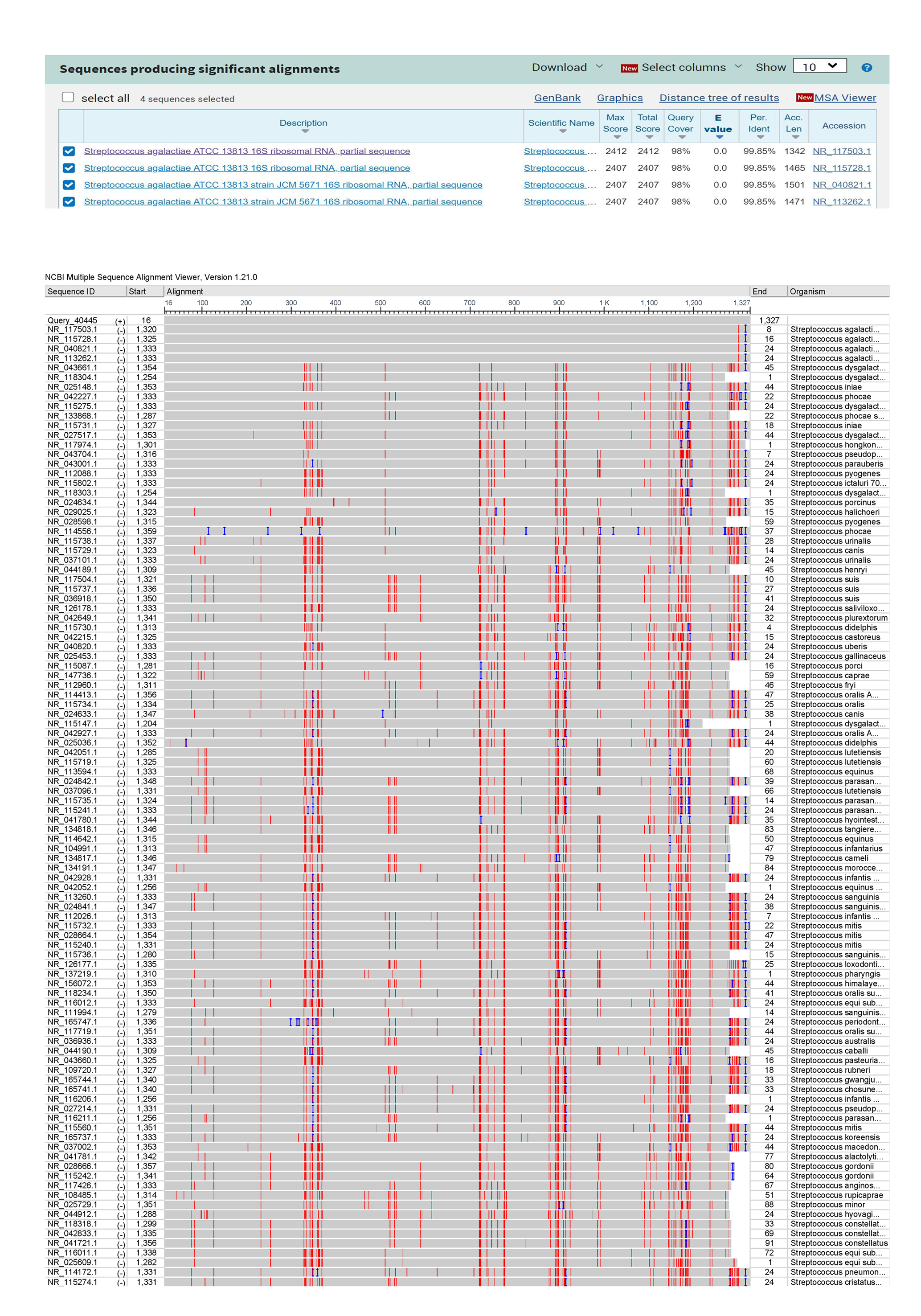

Supplement: Supplementary file 1 [file biomedicines-11-00097-s001.zip › Supplemental Figure S1.tif]

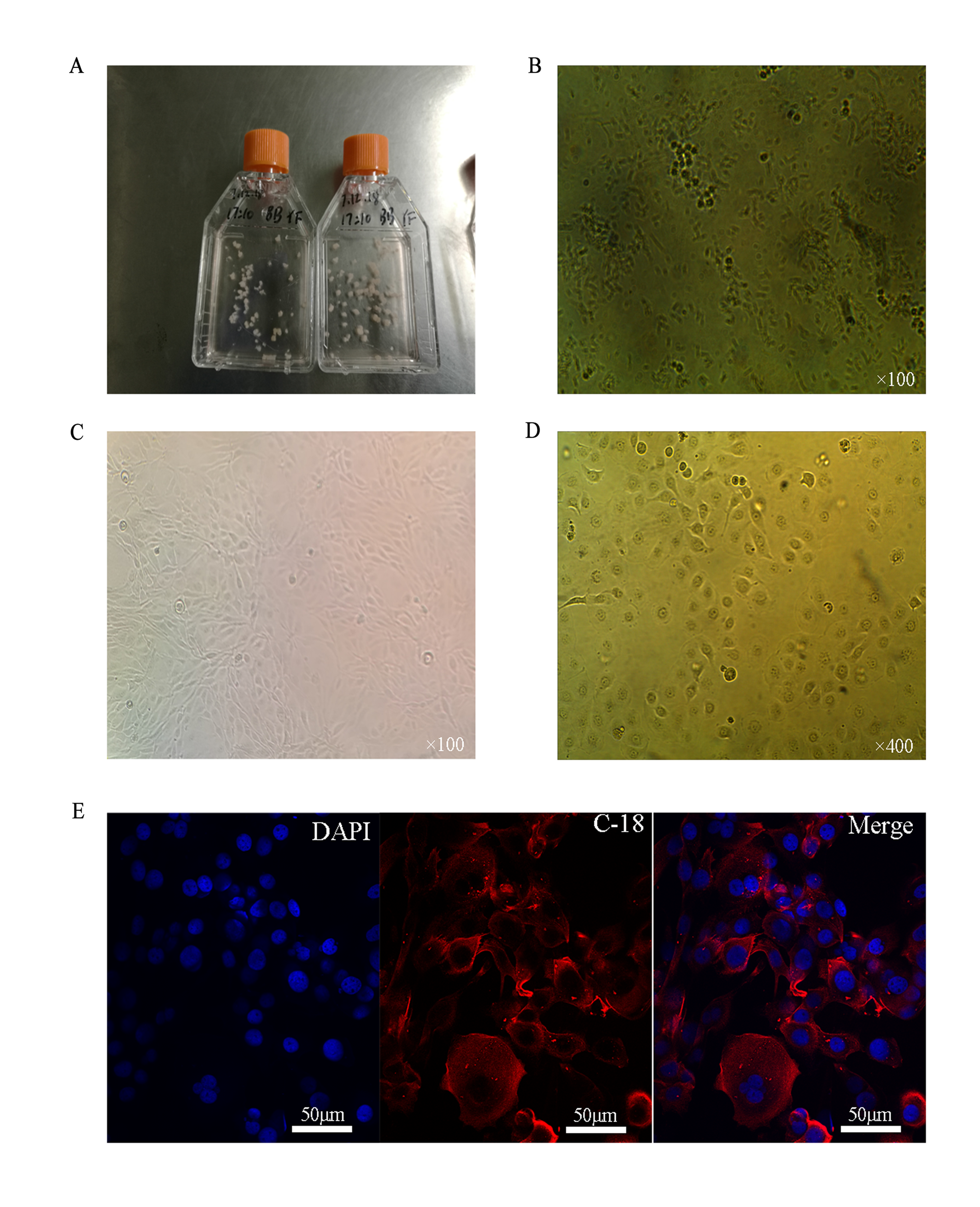

Supplement: Supplementary file 1 [file biomedicines-11-00097-s001.zip › Supplemental Figure S2.tif]

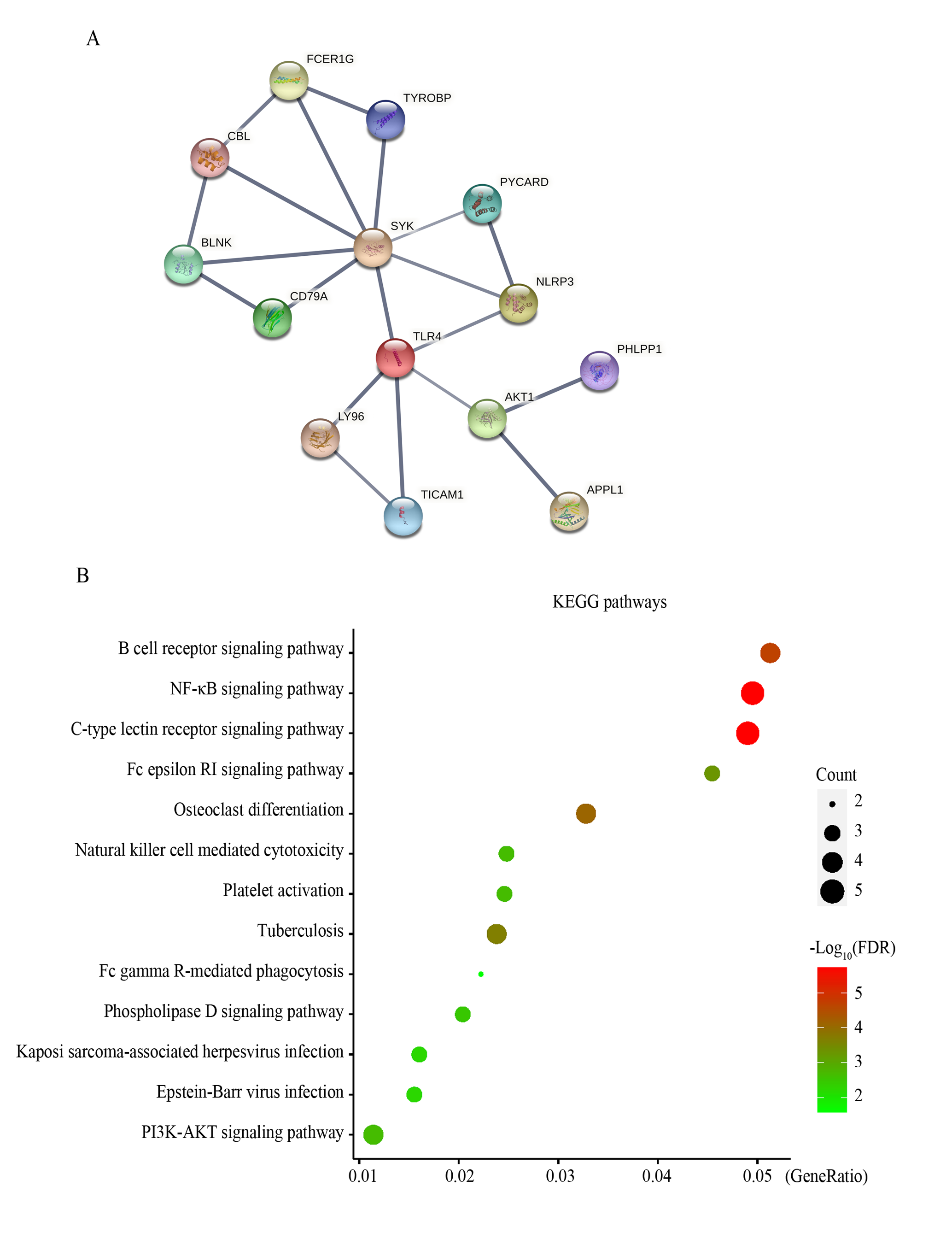

Supplement: Supplementary file 1 [file biomedicines-11-00097-s001.zip › Supplemental Figure S3.tif]

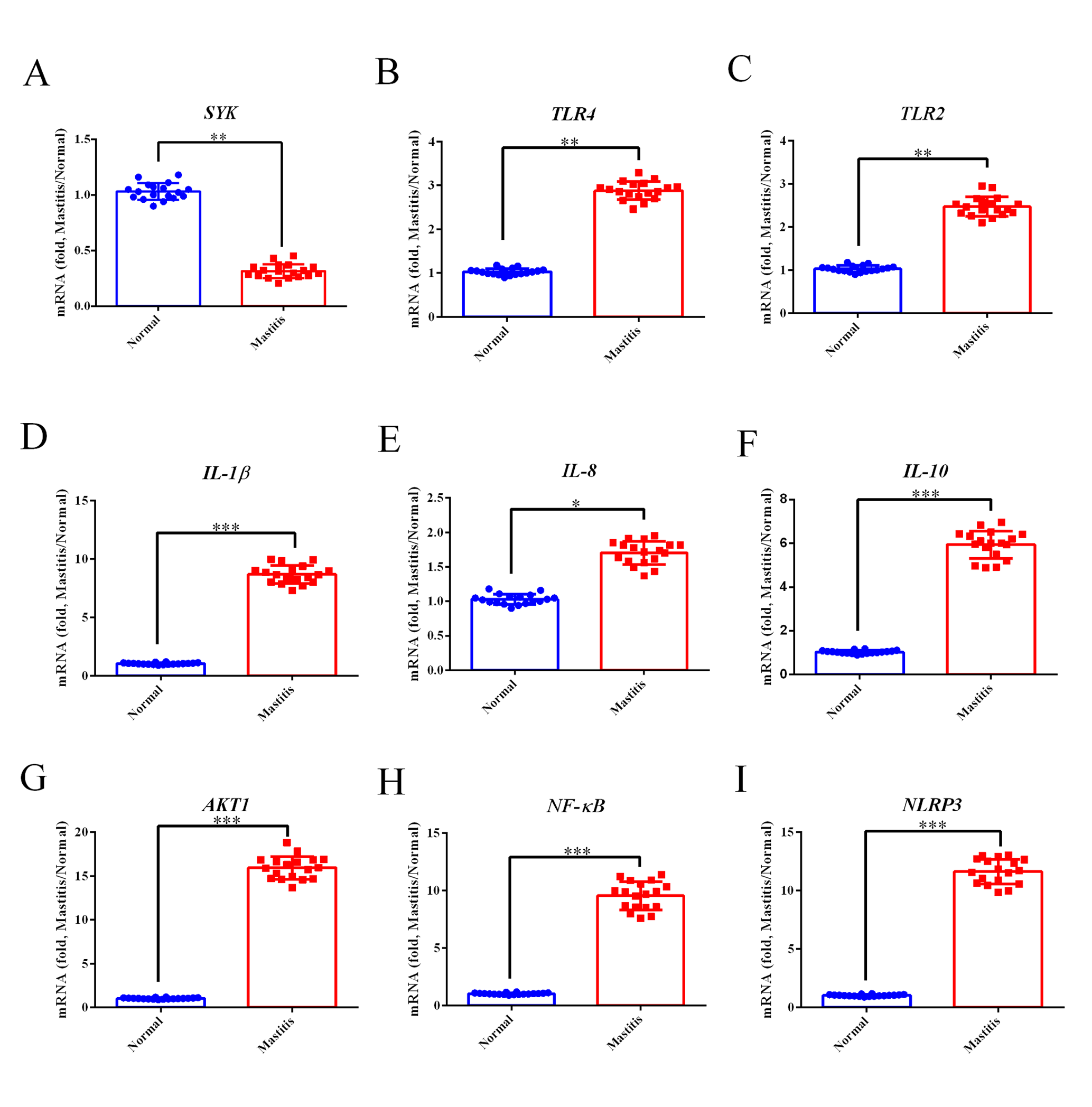

Supplement: Supplementary file 1 [file biomedicines-11-00097-s001.zip › Supplemental Figure S4.tif]

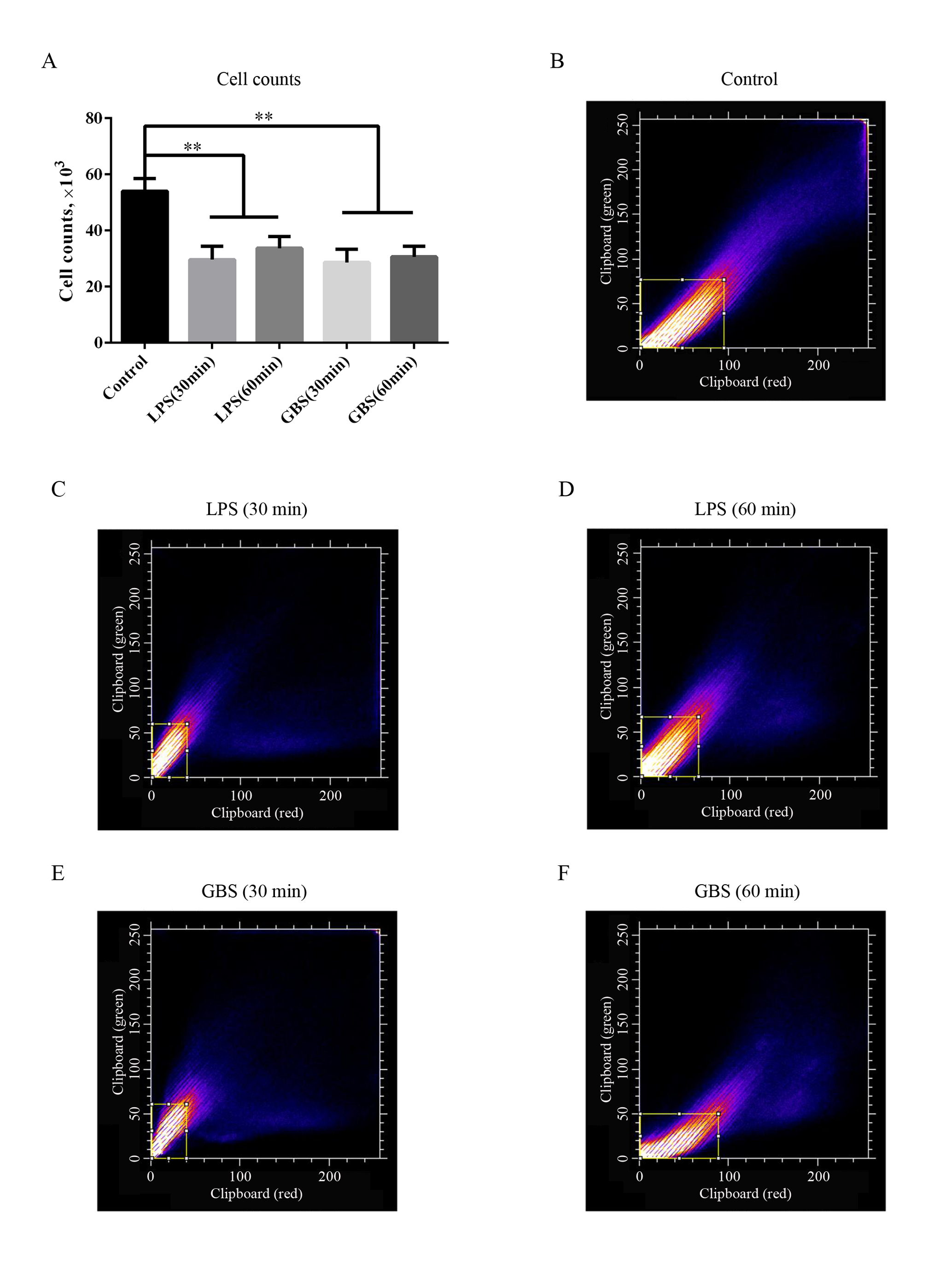

Supplement: Supplementary file 1 [file biomedicines-11-00097-s001.zip › Supplemental Figure S5.tif]

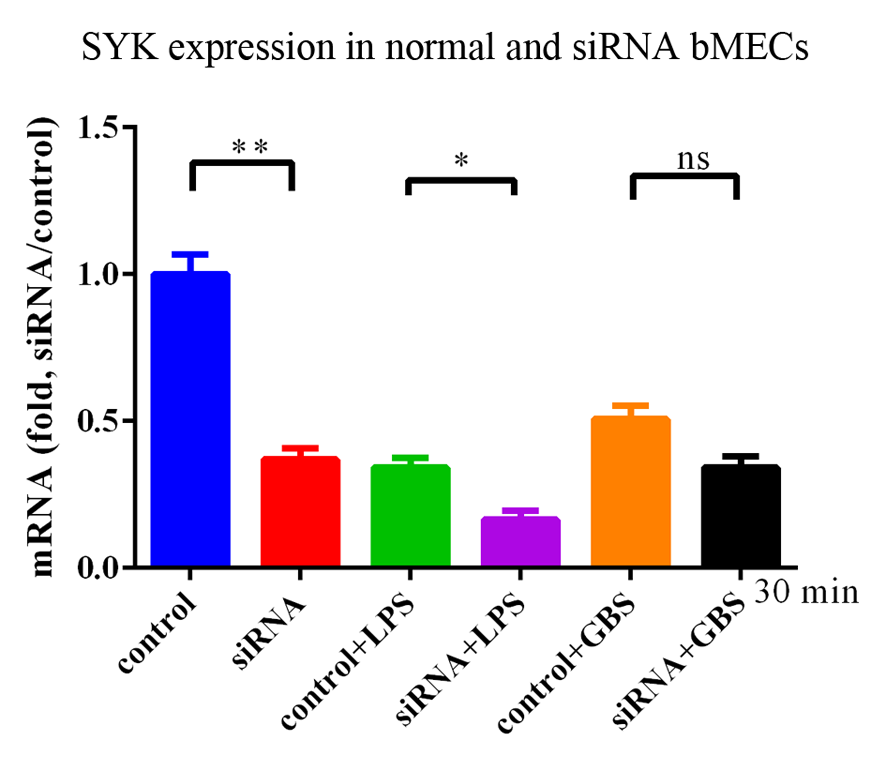

Supplement: Supplementary file 1 [file biomedicines-11-00097-s001.zip › Supplemental Figure S6.tif]
